# A Case of Central Venous Catheter-Related Bacteremia Caused by *Enterococcus gallinarum*

**DOI:** 10.1155/2023/9063371

**Published:** 2023-10-12

**Authors:** Ning Xu, Lei Zhu, Liyan Xiong, Jingjing Huo, Bin Wang, Xianyan Wu, Rui Tao, Qi Sa

**Affiliations:** ^1^Department of Clinical Laboratory, The First People's Hospital of Yunnan Province, The Affiliated Hospital of Kunming University of Science and Technology, Kunming, China; ^2^Department of Nephrology, The First People's Hospital of Yunnan Province, Kunming, Yunnan, China; ^3^Department of Reproduction, The First People's Hospital of Yunnan Province, The Affiliated Hospital of Kunming University of Science and Technology, Kunming, China; ^4^Sinopharm Group Yunnan Medical Equipment Co. Ltd., Kunming, China; ^5^Department of Hematology, The First People's Hospital of Yunnan Province, Kunming, Yunnan, China

## Abstract

A chicken farmer with cirrhosis and renal failure presented with an unusual case of catheter-related bacteremia. Testing with the VITEK® 2 Compact system, MALDI-TOF mass spectrometry, and 16S rDNA sequencing identified the pathogen as *E. gallinarum*. This case demonstrates the importance of maintaining a high level of contextual awareness in patients exposed to avian species to make an informed diagnosis and provide prompt treatment.

## 1. Introduction

Patients on maintenance hemodialysis (MHD) rely on vascular access for survival. Therefore, good vascular access is critical to hemodialysis. Due to its less invasive nature and easy access to rapid and high blood flow, the peripherally placed central catheter (PICC) is a staple instrument for establishing vascular access. However, since it is still an inherently invasive procedure, catheter-associated bacteremia, which can result in multiorgan failure, shock, and death [1], remains a valid and present concern [2]. In 1984, Collins identified *E. gallinarum*, an uncommon *Enterococcus*, from clinical samples as a Gram-positive coccus common in the digestive tracts of birds and animals [3]. In general, catheter-associated bloodstream infections are caused by *Staphylococcus aureus*, *Pseudomonas aeruginosa*, and coagulase-negative staphylococci; meanwhile, cases that involve *Enterococcus quail* are extremely rare. Enterococci have a complex resistance mechanism and are naturally resistant to cephalosporins, aminoglycosides (except for high levels of screening resistance), lincomycin, and cotrimoxazole. They are ineffective for clinical application even if they appear active in *in vitro* experiments [4]. Therefore, it is crucial that clinicians exercise critical thinking in selecting the correct antibiotics with a heightened level of contextual awareness.

## 2. Case Preparation

The patient is a 58-year-old man with cirrhosis who was initially admitted on July 25, 2021, to Yunnan Province's Xichu County Hospital due to pitting edema in both lower limbs and upper abdominal pain. The patient had a serum creatinine level of 1000 *μ*mol/L and was diagnosed with uremia and thrombocytopenia at the local hospital. He declined hemodialysis therapy during his stay but was discharged with a treatment plan for abdominal pain. On August 1st, 2021, the patient returned with black stool, increased edema, skin bruises on the lower limbs, wheezing, shortness of breath after exertion, an inability to lie in a supine position, and decreased urine output. The patient was admitted to our hospital with a fever of 36.0°C, a heart rate of 73 beats per minute, a blood pressure of 103/71 mmHg, a hemoglobin level of 81 g/L, and a platelet count of 46,109/L. A light microscope examination of renal tissue revealed diffuse mesangial proliferative glomerulonephritis and hepatitis B virus-related nephritis. The patient had indications of anemia, including scattered ecchymosis, ascites, and sunken edema in both lower limbs. He was diagnosed with stage 5 chronic renal failure and decompensated liver cirrhosis. On August 6, 2021, regular heparin-free dialysis therapy began, and a hemodialysis catheter was inserted beneath the left inguinal ligament. Mucosal and liver protection medication, diuresis and protein supplements, parenteral nutrition (RIVM), and symptomatic support were given to the patient.

**Figure 1:**
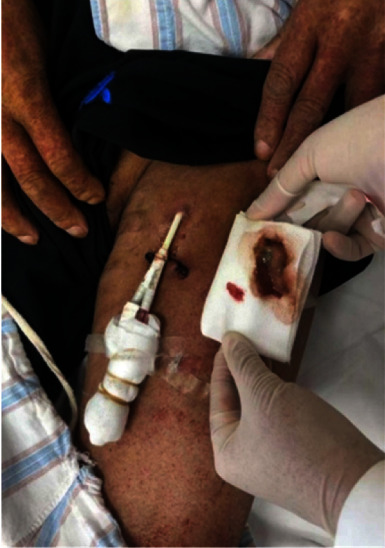
On the 4^th^ day of admission, the patient presented with chills, fever, and yellow pus visible as shown on the gauze and the outside of the catheter.

However, on August 10, 2021, the patient experienced chills and a fever, and yellow pus was visible on the outside of the catheter (Figure 1). The patient's body temperature was 38.9°C, heart rate was 96 bpm, blood pressure was 135/81 mmHg, and white blood cell counts were 11.63 × 109/L, with a neutrophil percentage of 80.5%, lymphocyte percentage of 16.7%, CRP of 67.20 mg/L, interleukin-6 of 40.50 pg/mL, and procalcitonin of 5.1 ng/mL. The infection was treated with cefmenoxime (1.0 g every 8 hours), and the deep venous catheter was flushed with cefazolin (2.0 g every 8 hours) and shut. On August 11, 2021, the patient's fever persisted, with a body temperature ranging from 38.9 to 39.2°C, and the infection index increased. The blood and wound secretions of the built-in dialysis catheter were sent for pathogen culture, and indomethacin was given to lower the temperature and provide physical cooling.

**Figure 2:**
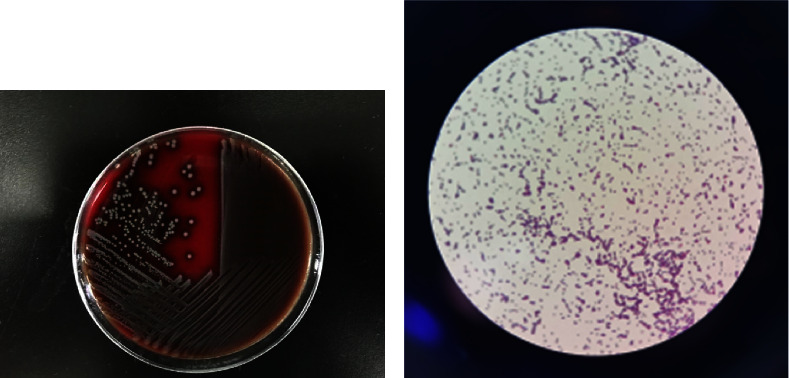
(a) The patient's blood culture was reported as positive for both aerobic and anaerobic vials. After 24 hours of incubation, the blood plate showed round, moist colonies of beige color. (b) The smear was stained for Gram-positive cocci.

**Figure 3:**
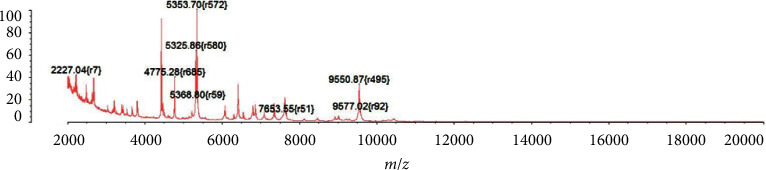
The matrix-assisted laser desorption/ionization time-of-flight mass spectrometer (bioMérieux, France) identified the pathogen as *E. gallinarum* in the mass spectrometry peak map with 99% identification confidence.

**Figure 4:**
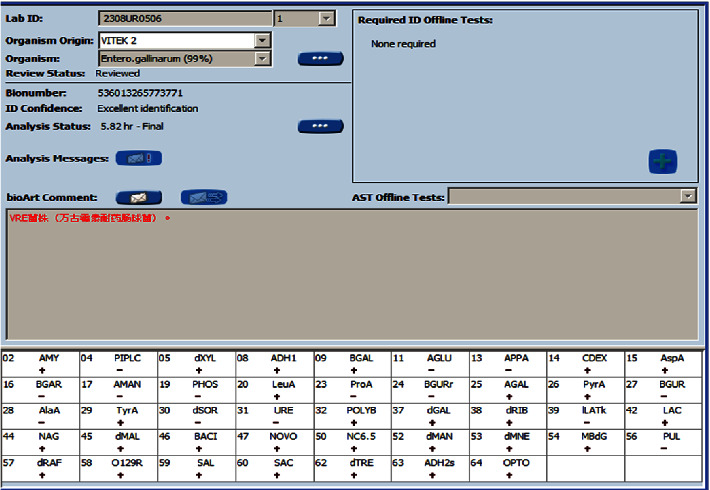
bioMérieux VITEK 2 biochemical ID results.

**Figure 5:**
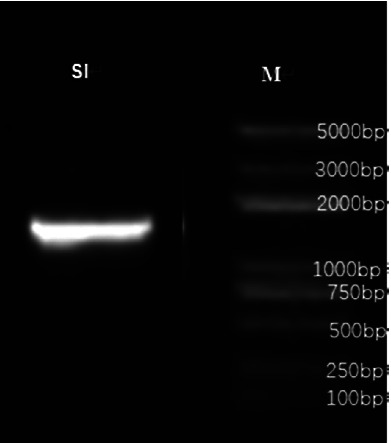
The 16S ribosomal DNA was amplified by using primers 27F and 1492R. The length of the amplification product was 1500 bp, and the Sanger method was used for sequencing. The gel sequence of the 16S ribosomal DNA gene sequence 100% matched *E. gallinarum.*

**Figure 6:**
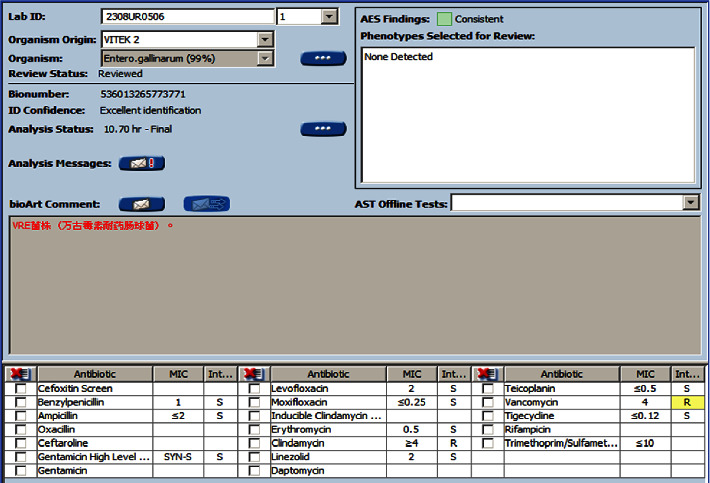
bioMérieux fully automatic microbial identification and drug susceptibility analyzer VITEK 2.

After 16 hours, both aerobic and anaerobic bottles of the patient's blood culture were positive. The blood plate showed off-white, spherical, moist colonies after 24 hours of incubation (Figure 2(a)). The Gram stain of the smear revealed cocci (Figure 2(b)). Both the catalase test and the motility test were positive. The VITEK 2 Compact automatic bacterial identification susceptibility tester (France bioMérieux) and matrix-assisted laser desorption/ionization time-of-flight mass spectrometry (France bioMérieux) identified the pathogen as *E. gallinarum* (ID confidence interval 99%; see Figures 3 and 4). For further verification, 16S ribosomal DNA gene sequence analysis was performed on the pure colonies. Amplicons were purified (Figure 5) and sequenced. In this study, the full-length sequence of 16S ribosomal DNA was amplified by PCR using specific primers 27F : AGAGTTTGATCCTGGCTCAG and 1492R : TACGGCTACCTTGTTACGACTT. The amplified fragment length was 1500 bp, and the amplified product was sequenced using the Sanger method. It was confirmed as *E. gallinarum* using NCBI BLAST. An evolutionary tree was plotted. (Sequences and the tree are available in supplementary materials.) At the same time, the results were compared using the basic partial alignment search tool on the database website of the National Center for Biotechnology Information (NCBI) (for sequence, see NCBI Seq: SUB13794671 Enterococci OR481706). The pathogen was again positively identified as *E. gallinarum*, with a comparison score of 100%. The results of the drug sensitivity test showed that the bacteria were sensitive to ampicillin, penicillin, linezolid, tigecycline, levofloxacin, teicoplanin, moxifloxacin, and erythromycin and resistant to vancomycin and clindamycin (Figure 6). According to the drug susceptibility test, the patient's treatment plan was adjusted to an intravenous penicillin drip (2.0 g every 8 hours) considering the patient's underlying conditions. On August 15th, 2021, on day 5 of admission, the patient's body temperature gradually returned to normal, and the two consecutive blood cultures were negative. The infection symptoms gradually resolved, and various indicators returned to their normal range. Subsequently, an arteriovenous fistula was placed in the forearm and no recurrence of infection occurred. The patient continued regular hemodialysis.

## 3. Discussion

*E. gallinarum* is an opportunistic pathogen that is rarely isolated in clinical specimens and even more rarely in human blood or bone marrow. Nosocomial infections are mainly caused by patients undergoing invasive surgery or immunosuppression therapy [5]. However, catheter-related bloodstream infections caused by *E. gallinarum* are relatively rare. At the time of admission to the local hospital, the patient had no fever, upper respiratory tract or neurological symptoms, or peritoneal inflammatory infection. We hypothesize that the *E. gallinarum* infection occurred via the skin and mucosal system through the hemodialysis intravenous catheter puncture. Dialysis patients have underlying illnesses and a weakened immune system, which increase their risk of opportunistic infection [6, 7]. Since *E. gallinarum* is a disease peculiar to poultry hosts, the patient's profession as a chicken farmer from a remote region with poor personal hygiene collectively induced colonization. Low immunoglobulin levels indicate poor humoral immunity, which also increases the risk of opportunistic infections [8]. Due to its vanC drug resistance gene, *E. gallinarum* is naturally resistant to vancomycin [9]. Furthermore, since this patient has chronic renal failure, the glycopeptide teicoplanin is not appropriate for usage, according to guidelines for the prevention and treatment of vascular catheter-associated infections and bloodstream infections [10]. Therefore, penicillin was administered instead. The patient's body temperature and inflammatory infection markers steadily decreased and returned to normal after treatment. Admittedly, this scenario is highly particular. First, this case features a vulnerable group with an underlying disease. In recent literature, *E. gallinarum* infections were all among immunosuppressant users or people with low immunity [11, 12], and the case's reference value for individuals without underlying disease is limited. Second, in terms of species identification resolution, the sequences obtained by 16S rDNA amplicon sequencing in this case are sometimes not annotated to the species level. However, if conditions permit, whole metagenome shotgun sequencing can be used in addition to accurately identify microorganisms to the species level or even the strain level. Further in-depth studies at the genetic and functional levels can also be performed.

## 4. Conclusions

In conclusion, *E. gallinarum* was confirmed to be the cause of the catheter-associated bacteremia, and the blind treatment with cefmenoxime and cefazolin deep venous catheter closure resulted in delayed treatment and exacerbation of the patient's condition. In future cases, we recommend following the FDA's guidelines for using linezolid for *E. gallinarum* infections if the patient has a history of ampicillin allergy [13]. Linezolid is a new oxazolone with antibacterial activity against Gram-positive cocci comparable to vancomycin. It is efficient against vancomycin-resistant enterococci and can significantly relieve clinical symptoms in patients. Meanwhile, increasing the rate of arteriovenous fistula dialysis and maintaining tight, regular maintenance of central venous catheters can aid in the reduction of enterococcal *E. gallinarum* infections. Early detection and diagnosis, as well as the correct use of antibiotics, are critical to achieving positive results.

## Supplementary Materials

Supplementary Materials The full-length sequence of 16S ribosomal DNA was amplified by PCR using specific primers 27F.
