# Supplementary material for: A Case of Central Venous Catheter-Related Bacteremia Caused by Enterococcus gallinarum
Source: Case Rep Infect Dis. 2023 Oct 12;2023:9063371. doi: 10.1155/2023/9063371 (PMC10656200; doi:10.1155/2023/9063371)
Supplement: Supplementary Materials — The full-length sequence of 16S ribosomal DNA was amplified by PCR using specific primers 27F. [file 9063371.f1.docx]

In this study, the full-length sequence of 16S ribosomal DNA was amplified by PCR using specific primers 27F: AGAGTTTGATCCTGGCTCAG and 1492R: TACGGCTACCTTGTTACGACTT, and the amplified fragment length was 1500 bp, and the amplified product was sequenced using the sanger method. The sequence was listed as follows：

CAGGACGAACGCTGGCGGCGTGCCTAATACATGCAAGTCGAACGCTTTTT

CTTTCACCGGAGCTTGCTCCACCGAAAGAAAAAGAGTGGCGAACGGGTGA

GTAACACGTGGGTAACCTGCCCATCAGAAGGGGATAACACTTGGAAACAG

GTGCTAATACCGTATAACACTATTTTCCGCATGGAAGAAAGTTGAAAGGC

GCTTTTGCGTCACTGATGGATGGACCCGCGGTGCATTAGCTAGTTGGTGA

GGTAACGGCTCACCAAGGCCACGATGCATAGCCGACCTGAGAGGGTGATC

GGCCACACTGGGACTGAGACACGGCCCAGACTCCTACGGGAGGCAGCAGT

AGGGAATCTTCGGCAATGGACGAAAGTCTGACCGAGCAACGCCGCGTGAG

TGAAGAAGGTTTTCGGATCGTAAAACTCTGTTGTTAGAGAAGAACAAGGA

TGAGAGTAGAACGTTCATCCCTTGACGGTATCTAACCAGAAAGCCACGGC

TAACTACGTGCCAGCAGCCGCGGTAATACGTAGGTGGCAAGCGTTGTCCG

GATTTATTGGGCGTAAAGCGAGCGCAGGCGGTTTCTTAAGTCTGATGTGA

AAGCCCCCGGCTCAACCGGGGAGGGTCATTGGAAACTGGGAGACTTGAGT

GCAGAAGAGGAGAGTGGAATTCCATGTGTAGCGGTGAAATGCGTAGATAT

ATGGAGGAACACCAGTGGCGAAGGCGGCTCTCTGGTCTGTAACTGACGCT

GAGGCTCGAAAGCGTGGGGAGCGAACAGGATTAGATACCCTGGTAGTCCA

CGCCGTAAACGATGAGTGCTAAGTGTTGGAGGGTTTCCGCCCTTCAGTGC

TGCAGCAAACGCATTAAGCACTCCGCCTGGGGAGTACGACCGCAAGGTTG

AAACTCAAAGGAATTGACGGGGGCCCGCACAAGCGGTGGAGCATGTGGTT

TAATTCGAAGCAACGCGAAGAACCTTACCAGGTCTTGACATCCTTTGACC

ACTCTAGAGATAGAGCTTCCCCTTCGGGGGCAAAGTGACAGGTGGTGCAT

GGTTGTCGTCAGCTCGTGTCGTGAGATGTTGGGTTAAGTCCCGCAACGAG

CGCAACCCTTATTGTTAGTTGCCATCATTTAGTTGGGCACTCTAGCGAGA

CTGCCGGTGACAAACCGGAGGAAGGTGGGGATGACGTCAAATCATCATGC

CCCTTATGACCTGGGCTACACACGTGCTACAATGGGAAGTACAACGAGTT

GCGAAGTCGCGAGGCTAAGCTAATCTCTTAAAGCTTCTCTCAGTTCGGAT

TGTAGGCTGCAACTCGCCTACATGAAGCCGGAATCGCTAGTAATCGCGGA

TCAGCACGCCGCGGTGAATACGTTCCCGGGCCT

It was confirmed as E. gallinarum by NCBI BLAST comparison.

NCBI was also compared and an evolutionary tree was plotted with the following results
